# Effects of Nautical Traffic and Noise on Foraging Patterns of Mediterranean Damselfish (*Chromis chromis*)

**DOI:** 10.1371/journal.pone.0040582

**Published:** 2012-07-11

**Authors:** Claudia Bracciali, Daniela Campobello, Cristina Giacoma, Gianluca Sarà

**Affiliations:** 1 Dipartimento di Scienze della Terra e del Mare, Università di Palermo, Palermo, Italy; 2 Dipartimento di Scienze della Vita e Biologia dei Sistemi - Life Sciences and Systems Biology, University of Turin, Turin, Italy; 3 Department of Environmental Biology and Biodiversity, University of Palermo, Palermo, Italy; National Institute of Water & Atmospheric Research, New Zealand

## Abstract

*Chromis chromis* is a key species in the Mediterranean marine coastal ecosystems where, in summer, recreational boating and its associated noise overlap. Anthropogenic noise could induce behavioural modifications in marine organisms, thereby affecting population dynamics. In the case of an important species for the ecosystem like *C. chromis*, this could rebound on the community structure. Here, we measured nautical traffic during the summer of 2007 in a Southern Mediterranean Marine Protected Area (MPA) and simultaneously the feeding behaviour of *C. chromis* was video-recorded, within both the no-take A-zone and the B-zone where recreational use is allowed. Feeding frequencies, escape reaction and school density were analysed. *C. chromis* specimens were also collected from 2007 to 2008 to evaluate their physiological state using the Body Condition Index as a proxy of feeding efficiency. The MPA was more exploited by nautical tourism during holidays than on weekdays, particularly in the middle of the day. Greater traffic volume corresponded with lower feeding frequencies. The escape reaction was longer in duration (>1 min) when boat passed nearby, while moored boats did not induce an escape response. We found no differences in density between schools in the A- and B-zones and worse body conditions among those individuals inhabiting the B-zone in one area only. Overall, our findings revealed a significant modification of the daily foraging habits of *C. chromis* due to boat noise, which was slightly buffered by no-take zones established within the MPA.

## Introduction

Nautical traffic has been recognized as a source of anthropogenic noise [1], [2] that can induce behavioural modifications in marine organisms [3]-[7]. Recreational boat noise generally displays frequencies below 1000 Hz [8] and many fish species can detect sounds from 100 to 1000 Hz [9]. Fish use biological sounds to obtain environmental information [10] and to recognize and communicate with conspecifics [11]-[15]. Human-produced sounds in the same frequency range of biological ones may mask the latter, with consequent repercussions on both behavioural and population dynamics [4]. Few studies have, however, been conducted in the natural environment or in semi-captivity [6], [16] to assess rebound on commercial species and fisheries [17]-[19] or marine mammals [3], [20], [21]. Furthermore, there are no studies across the current literature that have investigated the effect of human-produced sound on important habitat formers (ecosystem engineers, *sensu* [22]) such as the damselfish (*Chromis chromis*).

The damselfish is the most common and most abundant zooplanktivorous species in the marine coastal ecosystems of the Mediterranean Sea [23]-[25]. It drives faster nutrient and organic matter transfer from pelagic to benthic habitats through faeces production [26], [27]. Feeding behaviour is characterized by bimodal daily patterns - active feeding within a school in the middle of the water column in the daytime, and resting in hidden refuges at the bottom during the night [28]. Feeding activity is a function of light polarization [29], and is therefore variable during the day [30]. Foraging rates peak at midday when the greatest amount of light is available [31]. *C. chromis* could be considered as a key species for the ecosystem [32], where “key species” means “functional taxa without redundancy” whose loss or density changes could result in significant modifications to community structure. A primary consequence would appear to be shifts in the feeding rhythms and efficiency of damselfish, which in turn might affect dynamics of the matter (C, N and P) and energy fluxes through marine coastal communities. Monitoring this species is therefore crucial to understanding ecological processes in marine coastal environments.

Above all in summer, *C. chromis* schools are exposed to an intense and consistent volume of nautical traffic and, therefore, to the noise associated with the numerous recreational boats along coastlines [33], [34]. Ellison and colleagues [35] suggested a “three-part approach” to evaluate animal behavioural responses to the sound in which (i) exposure to different sounds, or sound levels, (ii) analysis of the relative sound levels, and (iii) exposure to acute and chronic sounds comprise their complementary analysis [35]. Codarin et al. [8] applied this approach in part when they showed that the noise produced by a medium-size boat reduced *C. chromis* auditory sensitivity in lab experiments. In our study, we aimed to use the whole approach proposed by Ellison et al. [35] by examining both acute responses to different noise sources and chronic responses to summer background noise increase, and their effects on the feeding behaviour of *C. chromis*.

The effect of diurnal boat noise on feeding behaviour may also have important repercussions on population functional response and, as such, on population dynamics. The resulting disturbance might induce modifications in foraging rates and patterns. Such changes may affect the amount of energy and time allocated by organisms to feeding which, in turn, is partitioned between food searching and handling [36], [37]. If boat noise is able to modify, quantitatively and qualitatively, any component of foraging budget allocation, then it would presumably also be able to affect *C. chromis*’ functional response and thereby their ultimate consumption rate [36].

Here, we verified whether nautical traffic and the associated noise affect the general feeding behaviour of the most abundant infralittoral fish of the Mediterranean Sea. Our specific goals were: (i) to quantify nautical traffic in tourist marine areas during summer at different times of the week and levels of MPA protection, (ii) to assess the effect of nautical traffic intensity on school behaviour, (iii) to estimate the feeding activity of *C. chromis* in terms of peak rate and timing of feeding behaviour and, lastly, (iv) to verify whether possible differences in feeding behaviour correspond to different body conditions of *C. chromis*. Other studies on anthropogenic sounds have assessed the effect of noise on predation risk (i.e. [38], [39]) but the implications of nautical noise on feeding habits and/or efficiency still remains a virtually unexplored field.

## Results

**Figure 1:**
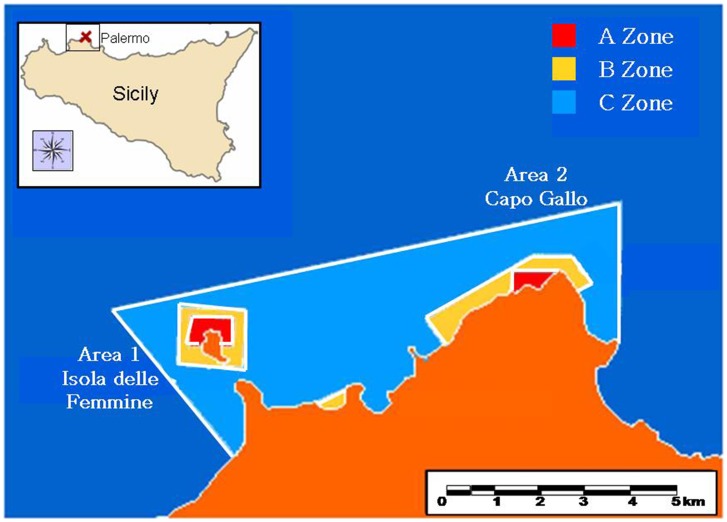
MPA of Capo Gallo and Isola delle Femmine, Southern Mediterranean, Italy. The map shows the Area 1 (Isola delle Femmine) and the Area 2 (Capo Gallo) that include A-zones (no recreational use allowed), B-zones (recreational use allowed), and C-zones (recreational use allowed together with fishing authorized by local authorities).

**Figure 2:**
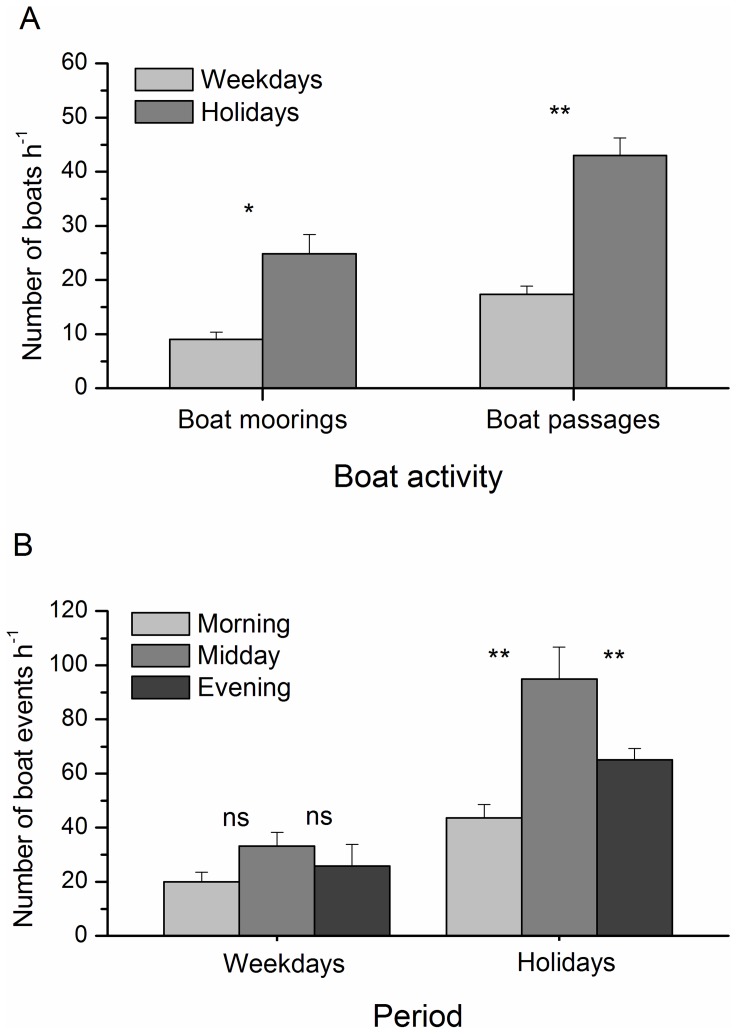
Nautical traffic intensity. Nautical traffic was monitored (A) between week periods (i.e. weekdays and holidays) and (B) throughout the day (i.e. morning, midday, and evening). The total number of boats was detected via visual census and it is presented as number of boats per hour. Error bars represent the standard error of the mean. The significance level is reported in figure: ns  =  not significant difference; * *P*<0.05; ** *P*<0.01. See text for ANOVA and post-hoc SNK test statistics.

Boat passages and total boat events were more frequent in Area 1 than in Area 2 (Fig. 1, ANOVA, Table 1, Table S1). In both areas, the number of boat passages, boat moorings, and total boat events were significantly different between periods, being lower on weekdays than holidays (Table 1, Fig. 2A). On weekdays, the total numbers of boat events were not significantly different among times of day (Table 1, SNK test, p>0.05), whereas they reached a significantly higher peak at midday during holidays (SNK test, p<0.001, Fig. 2B, Table S1). Mean (±SE) boat traffic variables are reported in Table S1. From these results we identified three significant different levels of traffic intensity: *low*, at any time of weekday; *medium*, including mornings and evenings on holidays; and *high*, including midday on holidays (Fig. 2B).

**Table 1:** ANOVA results of nautical traffic intensity.

*Boat passages* †				
Source	df	MS	*F*	p
Area	1	21.12	5.69	**0.0188**
Period (Per)	1	159.35	42.90	**<0.0001**
Time	2	12.89	3.47	**0.0346**
Per x Time	2	2.76	0.74	0.4779
Per x Area x Time	2	0.50	0.13	0.8748
Residuals	108	3.71		
***Boat moorings*** †				
**Source**	**df**	**MS**	***F***	**p**
Area	1	10.99	3.06	0.0831
Period (Per)	1	110.46	30.74	**<0.0001**
Time	2	43.64	12.14	**<0.0001**
Per x Time	2	2.85	0.79	0.4555
Per x Area x Time	2	0.13	0.04	0.9640
Residuals	108	3.59		
***Total boat events*** †				
**Source**	**df**	**MS**	***F***	**p**
Area	1	29.58	6.79	**0.0105**
Period (Per)	1	278.07	63.83	**<0.0001**
Time	2	46.10	10.58	**0.0001**
Per x Time	2	7.07	1.62	0.2020
Per x Area x Time	2	0.33	0.08	0.9268
Residuals	108	4.36		

† (squared +1) data transformed.

Nautical traffic intensity was measured as the number of boat passages, boat moorings, and total boat events recorded per hour in the different study areas, week periods and at different day times. Significant values are indicated in bold.

**Figure 3:**
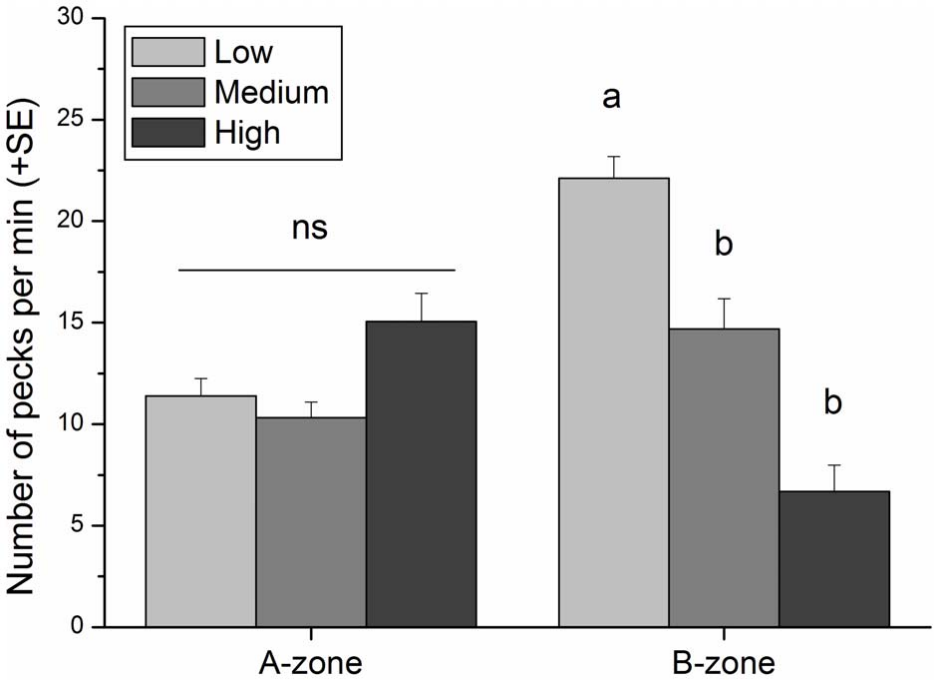
Pecking rate of *Chromis chromis related to nautical traffic intensity.* Pecking rate (±SE) of *C. chromis per minute* was recorded in A- and B-zones during periods of low, medium and high traffic intensities. Different superscripts indicate significant differences within zones resulted from pairwise SNK tests (Table 2) after ANOVA (*F*_2, 880_ = 20.64, *P*<0.0001). ns  =  not significant difference.

**Figure 4:**
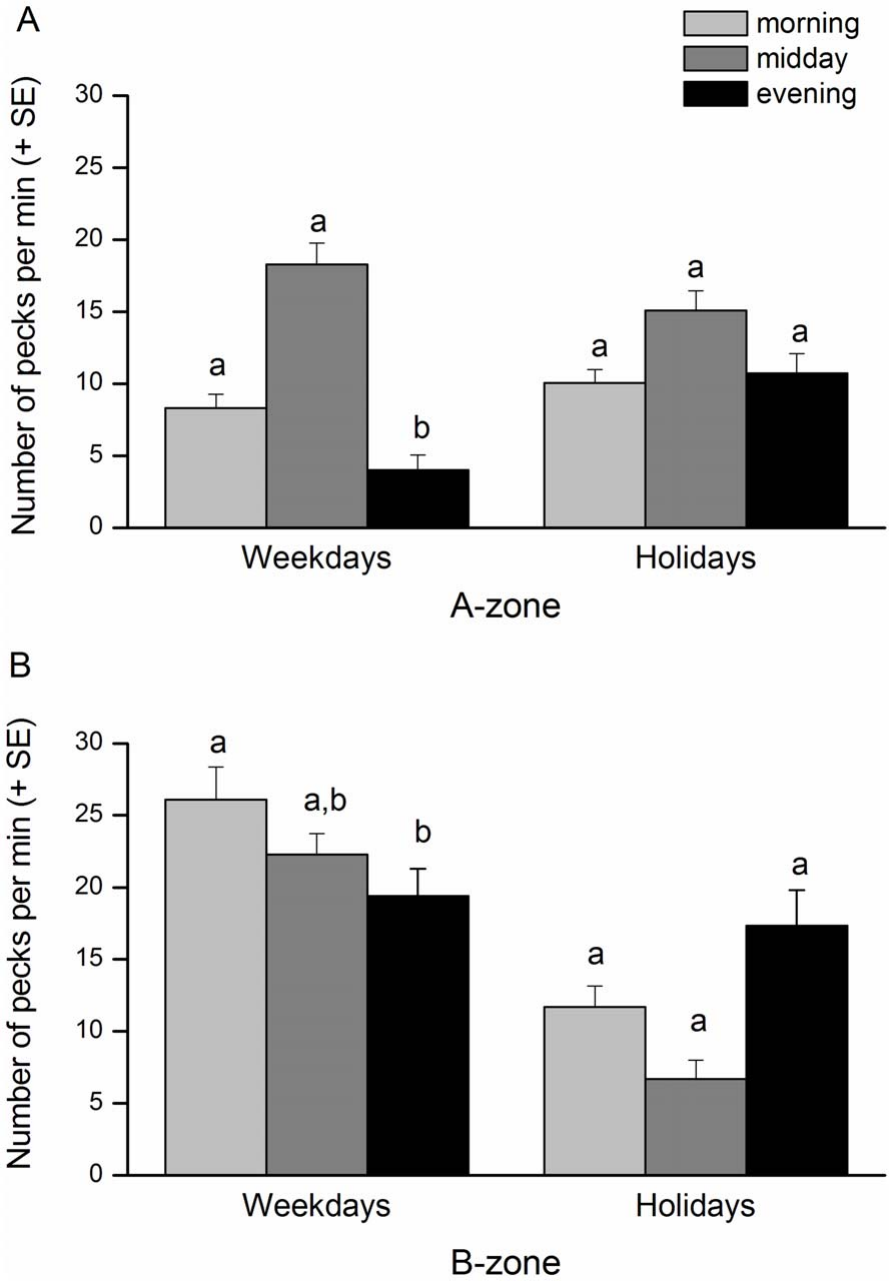
Analysis of pecking rate of *Chromis chromis* during the week. Pecking rate min^−1^ (±SE) of *C. chromis* was recorded in A- and B-zones, during weekdays and holidays, and in three time slots. Different superscripts indicate significant differences among the time slots, within periods (i.e. weekdays or holidays) and within zones resulting from pairwise SNK tests (Table 3) after ANOVA (*F*_2,874_ = 9.84, *P*<0.0001).

Foraging rate was not different between zones (ANOVA, *F*_1,880_ = 0.91, p = 0.3394, Table S2). Pecking rate was unaltered in the A-zone, whereas it was significantly lower in medium/high than low traffic intensity in the B-zone (Table 2, Fig. 3). Comparing pecking rates between zones and within traffic intensity level, *C. chromis* pecking rate in the B-zone decreased significantly during highly intense traffic, but significantly increased when nautical traffic was less intense (SNK tests, Table 2, Fig. 3). The least disturbed site (A-zone) during the period of lowest intensity of traffic (weekdays) showed a daily fluctuating foraging activity, in which *C. chromis* preferred pecking during the first part of the day, but almost stopped as daylight faded in the evening (ANOVA, *F*_2,874_ = 9.84, p<0.0001, Table 3, Fig. 4A). During holidays, although foraging at midday did not significantly decrease, fish increased their activity in the evening - pecking rate was therefore higher than on weekday evenings (Table 3, Fig. 4A). In the A-zone generally, foraging rate did not change between weekdays and holidays (Table 3, Table S2). In the B-zone, there was a completely different daily foraging pattern. On holidays, *C. chromis* inverted their activity intensity by increasing foraging in the evening and decreasing it at midday. Their pecking rates, however, did not show a significant difference among the three time slots (Table 3, Fig. 4B). Fish did significantly increase their foraging frequency on weekdays (ANOVA, *F*_1,874_ = 45.50, p<0.0001, Table S2), especially until midday (Table 3, Fig. 4B). Cumulatively in the two zones, pecking rate was significantly lower during holidays than weekdays (ANOVA, *F*_1,874_ = 14.83, p = 0.0001, Table 3, Table S2).

**Table 2:** Pecking rate under different traffic conditions.

	A low	A medium	A high	B low	B medium	B high
A low		0.7778	0.0540	**<0.0001**	0.4776	0.1196
A medium			0.0466	**<0.0001**	0.5817	0.0914
A high				**0.0085**	0.1089	**0.0002**
B low					**<0.0001**	**<0.0001**
B medium						0.0370

Differences in number of pecks min^−1^were recorded in A- and B- zones and during low, medium and high traffic intensities. *P*-values of SNK tests followed ANOVA (*F*_2, 880_ = 20.64, p<0.0001) are shown. Bold format indicates significant difference (α = 0.01).

**Table 3:** Pecking rate during the week in A- and B-zones.

	A, weekdays,MD	A, weekdays, PM	A, holiday, AM	A, holiday, MD	A, holiday, PM	B, weekdays, MD	B, weekdays, PM	B, holiday, AM	B, holiday, MD	B, holiday, PM	A,holidays	B, weekdays	B,holidays
A, weekdays, AM	0.0225	**0.0005**	0.9577										
A, weekdays, MD		**<0.0001**		0.4684									
A, weekdays, PM					**0.0006**								
A, holiday, AM				0.1218	0.8122								
A, holiday, MD					0.1041								
B, weekdays, AM						0.6052	**0.0229**	**0.0003**					
B, weekdays, MD							0.0631		**<0.0001**				
B, weekdays, PM										0.0501			
B, holiday, AM									0.2800	0.5099			
B, holiday, MD										0.5939			
A, weekdays											0.2673	**0.0000**	
A, holidays													0.3171
B, weekdays													**<0.0001**

Differences in the number of pecks min^−1^were recorded in A- and B- zones, during weekdays and holidays, and in three time slots. Time slots: morning (AM), midday (MD), and evening (PM). p -values of SNK tests followed ANOVAs (see text for statistics) are shown. Bold format indicates significant difference (α = 0.01).

**Figure 5:**
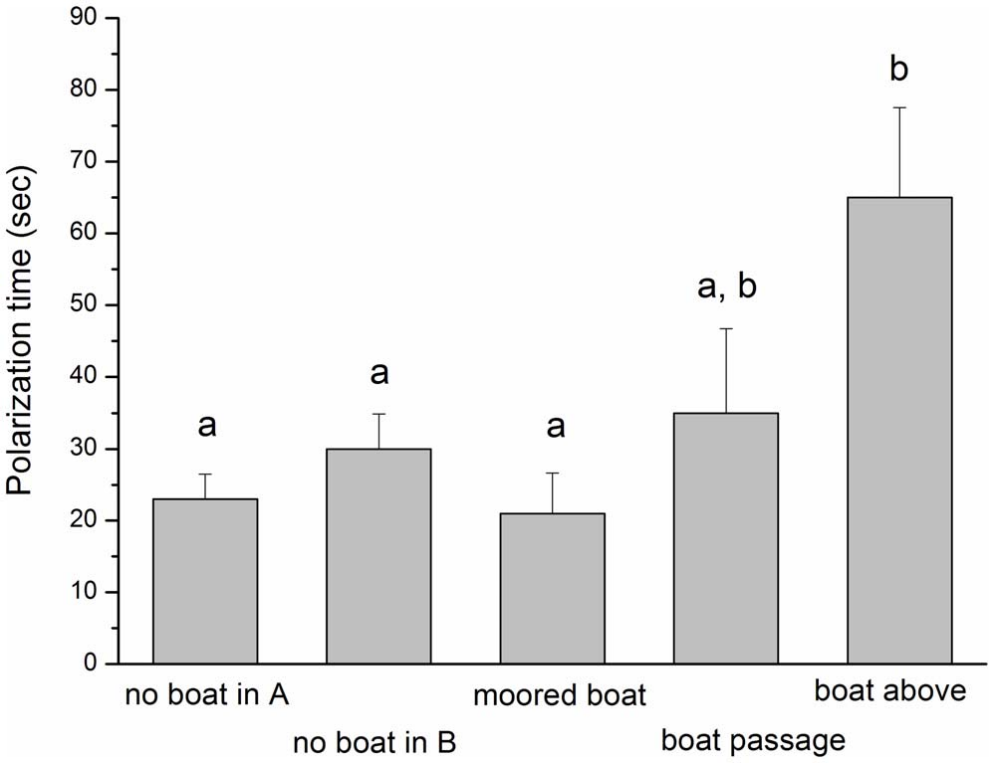
Nautical traffic induces polarization reaction in *Chromis chromis*. Polarization is a generic defensive behaviour and we monitored this event in the absence and in the presence of different types of boat. Polarization times are expressed in seconds. Error bars represent the standard error of the mean. Different superscripts indicate significant differences resulted from pairwise tests after PerMANOVA (Table 4).

Modifications of foraging activities were significantly longer when boats passed over the school, and within a 100 m radius of it (PERANOVA, Table 4, Fig. 5). Despite the recorded polarizations, we recorded school densities were not affected by nautical traffic and remained uniform under different traffic levels (PERANOVA, pseudo-*F* = 0.6709, p (perm) = 0.5669) with a mean density of 247.19 (±18.30 SE) individuals per video shot.

**Table 4:** Analysis of school polarization times.

Source	df	MS	Pseudo-*F*	p(perm)
Type of boat presence	4	13.31	4.6394	**0.0023**
Residuals	76	2.87		
Pairwise comparisons	*t*	p (perm)		
no boat A vs B	0.6112	0.5520		
no boat A vs moored boat	0.2973	0.7669		
no boat A vs boat passage	1.2239	0.2279		
no boat A vs boat above	4.2068	**0.0006**		
no boat B vs moored boat	0.6495	0.5288		
no boat B vs boat passage	0.9179	0.3787		
no boat B vs boat above	3.8865	**0.0009**		
moored boat vs boat passage	1.0751	0.2935		
moored boat vs boat above	3.7432	**0.0089**		
boat passage vs boat above	1.9354	0.1005		

PerMANOVA results showing the effect of different types of boat presence on the school polarization times of *C. chromis*. Bold format indicates significant difference.

**Figure 6:**
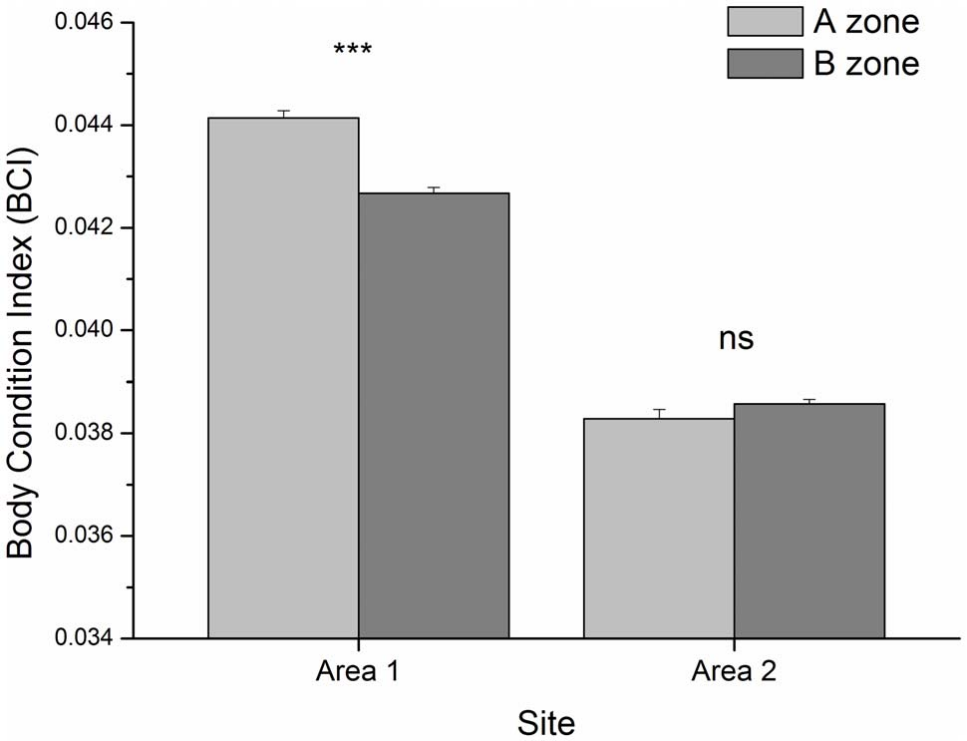
Body Condition Index (BCI) of *C. chromis*. *C. chromis* individuals were collected in periods of reproductive inactivity and the BCI was compared between zones in the two study areas. The BCI is equal to TW/SL^3^, where TW is Total Weight (0.1 g), and SL is Standard Length (0.005 cm), and shows the conditional state of a fish for a given length. Error bars represent the standard error of the mean. The significance level is reported in figure: ns  =  not significant difference; *** *P*<0.001 after ANOVA (see text for statistics).

In general, the mean value of BCI was higher for *C. chromis* living in the A- than in the B-zone (ANOVA, *F*_1,5294_ = 17.9, p<0.0001, SNK test, p<0.0001). This result was due to the difference in Area 1 (ANOVA: *F*_1,5294_ = 39.8, p<0.0001, SNK test: p<0.001) while the BCI was similar in the two Area 2 zones (SNK test: p = 0.1412, Fig. 6). Cumulatively in the zones, BCI values in Area 1 were higher than those in Area 2 (ANOVA: *F*_1,5294_ = 1271.8, p<0.0001, SNK test: p<0.0001).

## Discussion

Nautical tourism was a notable source of environmental disturbance in the studied MPA. The volume of nautical traffic remained even in the MPA throughout the study period, with peaks at weekends. Specifically, holidays presented a traffic volume exceeding that of weekdays by 2–3 times, with a peak of 90 boat events per hour at midday. The maximum level of traffic detected in this area is comparable to that of large harbours such as Sydney (Australia, [40]). From June to September, nautical traffic fluctuated cyclically, with periods of low (i.e. weekdays) and high (i.e. holidays) intensity, to which *C. chromis* adjusted their feeding frequency. Repeated noisy conditions over time provided us with optimal experimental field settings [35] for carrying out observations at different temporal scales. Variations in environmental sound due to low and intense traffic occurring over short (i.e. daily) and medium (i.e. weekly) periods allowed us to record chronic foraging responses, while single disturbance events, such as boat passages, enabled us to record acute responses of *C. chromis* to different noise sources.

### Behavioural Modifications of *C. chromis*

Pecking rates of *C. chromis* were strongly affected by nautical traffic and boat noise. An increase in nautical traffic was followed by significantly less frequent foraging activity events whereas low traffic intensity was significantly associated with an increase in pecking rates. Noise induces similar behavioural responses in other fish species [41] and in marine mammals [21] where individuals modify their behaviour in accordance with the environmental pressure [42]. Possible mechanisms of this change are that noise diverts organisms’ attention [41] or induces escape reactions [21], so that individuals are forced to maximize food intake at times when this element is absent from their environment. Drivers of this behavioural adaptation are probably both physiological and cognitive. Lost foraging opportunities during hostile conditions may be recovered and driven by the need, on the one hand, to feed [43], [44] and, on the other, by the “memory of past day’s feeding history”, thus on the past day’s food availability [45]. Increasing their pecking rates significantly during low traffic intensity allowed Mediterranean damselfish to quantitatively compensate for the reduced foraging efficiency experienced during noisier days or hours.

Although foraging adjustments did still permit a certain level of pecking efficiency, as recently shown in three-spined sticklebacks (*Gasterosteus aculeatus*, [41]), the daily feeding pattern of *C. chromis* was significantly modified in the B-zones at the highest levels of traffic, with foraging peaking at sunset. This was an inverse pattern to the natural one where, as shown in no-take A-zones with little traffic, *C. chromis* foraged intensely during the day, when sunlight enabled better detection of their zooplankton prey [30]. Although traffic increase in the A-zones did not affect the overall foraging rate we nevertheless recorded a slight effect, with a pecking rate decrease at midday and an increase in the evening. This indicated that the A-zone was still able to function both as a buffer and a thin barrier against the heavy traffic disturbance coming from the B-zone. Such a fact can have important implications for the management of marine natural resources and for setting boundaries of no-take zones in MPAs [46].

### Possible Cause of Changed Foraging Patterns

Polarization is a generic defensive behaviour, where individuals temporarily abandon the feeding patch by moving towards the bottom [47]. Foraging patterns of *C. chromis* were modified by nautical traffic, particularly by moving boats that induced schools to polarize. The passing of boats from directly over the school within a 100 m radius resulted in significantly prolonged polarizations (up to 1 min each). Conversely, the presence of moored boats (motor switched off) produced brief polarizations similar to those recorded with background noise in the absence of any marine vessel, as with *Tursiops truncates*, whose behaviour was not affected by sailing boats [48]. Both results, i.e. presence and absence of polarizations in case of moving and moored boats respectively, indicate the noise generated by boats as a plausible factor inducing damselfish polarization.

Startle responses depend on the species-specific sound level threshold, which in turn depends on species-specific hearing sensitivity [49]. Hearing sensitivity of a generalist hearing species (e.g., *Oncorhynchus mykiss*) such as *C. chromis* [13], [50], was altered at low frequency exposure, showing a shift in the hearing threshold [51], but not at high frequencies, that instead affected hearing abilities of a specialist hearing species (*Ictalurus punctatus*) [52]. Boat noise was already known to reduce the auditory sensitivity in *C. chromis* [8] relative to the perception of conspecific vocalizations, but no studies have been conducted to date that determine their sound threshold for startle responses. Several studies clearly indicated that startle responses in fish are induced by an initial acoustic stimulus rather than continuous exposure to sound [53]-[56]. Fish can adapt to ambient noise [55], [57] but abrupt changes in sound characteristics, such as those caused by the passage of a boat, induce rapid bursts of swimming activity [6], [54]-[56] away from the sound source [17], [18], [58]. In general, this corresponds with the frequent polarizations we recorded as boats passed over or near the schools.

### Potential Effects of Changed Foraging Patterns

The temporary loss of the feeding patch, i.e. polarization, is an energy consuming process. At peak nautical traffic times *C. chromis* abandoned feeding patches for up to 1 min, with a frequency of about 30 polarizations per hour. When a fish is “forced” to move away from its original position, the interruption in foraging activity results in a reduced energy intake. It also has to *expend* energy to escape and increase its swimming speed in order to reach the bottom quickly [6], [18], [59]. While we assume that, in this species, the handling of food is negligible, we argue that with *C. chromis*, nautical traffic might affect the amount of time dedicated to food searching, with the inevitable consequence of a decreased food ingestion rate. Searching and ingestion rates are two competing functions with potential rebounds on energy flow at individual levels, which can potentially affect population dynamics [60]. Bioenergetic considerations [37], in terms of changes in functional response [36], [61], indicate that ultimate fitness of damselfish may become compromised under noisy conditions. Furthermore, if boat noise induces deviation from the habitual searching/ingestion relationship, a reduction in the maximum attainable size may be expected due to the reduction of incoming energy derived by the total food uptake.

Despite potential alterations in energy use patterns during feeding phases, the behavioural effects of polarization allow *C. chromis* to benefit from abandoning its position in the water column when fleeing from a stress factor, as in so doing, it approaches the relative refuge of the bottom. The benefit lies in the resulting lack of school dispersal and the correspondingly faster school re-formation when the stressor ceases [62]. The ability to reach the bottom easily and to then quickly recover the original position in the water column is suggested as a mechanism to increase population robustness and resilience [63], because it prevents individuals from permanently abandoning the site they inhabit. This response likely increases the ability of *C. chromis* to tolerate varying conditions and could be a key factor in explaining why nautical traffic exerts a negative effect on damselfish foraging behaviour, but not on its density. However, this hypothesis needs to be tested through large field surveys in other environmental contexts.

Although polarization serves as a good trade-off to optimize contrasting demands (i.e. feeding versus escaping), this defensive response is still a costly reaction in terms of energy, and probably leads to chronic stress [35]. This is because the action of escaping from the position gained in the water column due to boat movement is paid for, from a bioenergetic point of view, in terms of somatic maintenance costs [60]. Somatic maintenance is a competing function with growth and this possibly explains why, overall, the Body Condition Index of *C. chromis* appeared to be negatively affected by nautical noise. Although the entire MPA area has generally been described with regard to homogeneous geomorphology, temperature, salinity and chlorophyll [64], at smaller scale, geomorphologic diversity, temporal fluctuations and spatial differences of zooplankton available within the two study areas may have been responsible for both the differences and similarities of the body condition between the two zones in the two study areas. Future research efforts are required to assess whether the altered foraging pattern induced by boat traffic has a real biological impact on the body condition of foraging damselfish.

### Concluding Remarks

Nautical traffic and its associated noise disturbance recorded in this Mediterranean MPA significantly affected the foraging pattern of *C. chromis*. Zooplankton pecking rates decreased on days with heavy traffic, and foraging activity was significantly modified by nearby boats passages. The restrictions in the A-zones of the MPA were sufficient to ensure avoidance of most negative effects on foraging activity although, on busy days, these areas seemed scarcely able to buffer noise disturbance deriving from B-zones. The fish in worse condition were those found in the busiest zone, although this was true only in one of the two areas studied. We found significant different feeding patterns between *C. chromis* populations living in areas where recreational boats were allowed and the no-take zones within the studied MPA. One of the possible effects of nautical disturbance is apparent in behavioural modifications [65]. We hypothesize that these have a direct effect on two main components of *C. chromis* energy budgets: the relationship between ingestion and searching, and the somatic maintenance function. This hypothesis warrants further testing in the form of targeted lab and field studies with different species, to assess whether the balance between energy intake and energy expenditure from metabolic machinery is a possible key factor in explaining effects of nautical disturbance on individual fitness and population dynamics.

## Materials and Methods

### Ethics Statement

We obtained all necessary permits for the field study described here. The local Coast Guard, the legal authority responsible for the Marine Protected Area “Capo Gallo and Isola delle Femmine”, solicited and funded our study (DINAUTIS project). Field activity protocol, including sampling in the restricted A zones, was therefore authorized by registration no. 26 - 5/2/2008 sent to Admiral V. Pace, Captain of the local Coast Guard. No other permit was necessary as damselfish is not an endangered or protected species.

### Study Area

We conducted this study in the Marine Protected Area (MPA) of Capo Gallo and Isola delle Femmine (Northwest Sicily, Italy), in July and August 2007 and 2008, to determine the effects of nautical traffic on *C. chromis* dynamics. The MPA is an area of approximately 20 km^2^ with three zones of different levels of protection (Fig. 1): the A-zone, a no-take area where no recreational use, including diving and fishing, is allowed, the B-zone, where recreational use is allowed, and the C-zone, where recreational use is allowed as well as fishing with permits from the local authorities. Recreational nautical traffic included activities such as boat passages and mooring within buoy-fields. Our study site comprised two areas: Isola delle Femmine (Area 1) and Capo Gallo (Area 2, Fig. 1), both northward-oriented and with similar geomorphology (rocky-vegetated substratum in crevices alternated with sandy bottom covered by the seagrass *Posidonia oceanica*). The mean depth was about 15 m with rare instances of 40/50-m depths (www.ampcapogallo-isola.org). Within the same area, at depths of 3 m from the surface, the A- and B-zones were characterized by similar temperature (∼ 24 C°), salinity (∼37.6±0.7) and chlorophyll (∼0.65 µg L^−1^) values (DINAUTIS 2009; [64]). According to the different area and level of activity restrictions, we defined four study sites: A1 and B1 at Isola della Femmine (Area 1) and A2 and B2 at Capo Gallo (Area 2, Fig. 1).

### Sample Design

To determine whether traffic intensity was distributed differently across periods in the week and times of day, and to test the effect of nautical traffic on *C*. *chromis* behaviour, we simultaneously sampled both nautical traffic and *C. chromis* behaviour during holidays and weekday periods and in three different time slots [20]: morning (8:30–10:30 a.m., Central European Time), midday (12:30–2:30 p.m.), and evening (5:00–7:00 p.m.).

### Nautical Traffic

We measured nautical traffic in B1 and B2 sites (A-zone was an off-limits zone) from four fixed stations located on the coast, where operators recorded the total number of boats passing by or mooring. We also quantified nautical traffic during holidays and weekdays, repeated five times for each. Samplings were carried out during three time slots, where each session lasted 15 minutes and were replicated four times per time slot. We defined nautical traffic intensity as the number of boats per hour.

### Behavioural Analysis

We sampled behaviour of *C.* *chromis* schools living in the water column of maximum 12 m depth. A SCUBA diver filmed *C. chromis* with a SONY video camera equipped with a NIMAR housing. Recording started 10 minutes (min) after the diver arrived at the site, and lasted 40 min. For the first 20 min, divers filmed at 10 m from the school, and for the remaining 20 min they approached individual *C. chromis* at 1–2 m.

#### Video analysis

As part of the acclimatization protocol, we did not analyze the first 5 min of video recordings and other 5 min between school and individual samplings [66]. From the remaining 30 min, we dedicated the first 15 min to school analysis and the last 15 min for individual analysis, as follows:

#### School analysis

School analysis aimed to quantify two variables: a) school density and b) school polarization time. We determined school density by dividing video-recordings into three 5-min frames, from which we took three 22×16-cm random shots. From each shot, we determined the percentage of space occupied by the school and relative *C. chromis* abundance by using a 2×2 cm-square grid mounted on a PC screen. Thus, we quantified *C. chromis* relative density as the number of individuals per shot and on a two-dimensions plane only. We chose this last method to render the density evaluation homogeneous and possible by counting individuals whose length was 5±1 mm.

School polarization is the defensive behaviour that occurs when all school members stop feeding, swim simultaneously toward the bottom, and keep both the caudal fin with a reduced opening and the pectorals close to the body [6], [47]. Polarization ends when all members spread out again in the water column, recover the original random orientation and resume feeding. From videos we observed polarizations that took place under four different conditions: 1) no boat, with apparent boat absence, 2) moored boat, with motor turned off and located straight above the schools, 3) boat passage, with boats passing at 50–100 m from the school, and 4) boat above, with boats passing right above the school. To validate these four conditions, we synchronized our underwater recordings with those of two operators on a boat, who recorded the presence of moored boats or boats passing above the observation site or at 50–100 m away from it. We counted the duration of each polarization by using JWatcher 1.0 software [67].

#### Individual analysis

From the central 10 min of an individual recording, we randomly selected a frame of 15 s within each min. Within each frame, we counted the feeding events of *C. chromis* as the number of pecks (i.e. mouth opened, put forward and then back closed) that we converted into individual pecking rate (peck min^−1^).

### Biometrical Analysis

*C. chromis* specimens were collected from the four study sites using a circular net (50×6 m) manoeuvred from a small fishing boat. The experimental catches took place during October 2007, and monthly from April to November 2008. Given the gonadic influence on growth rate [60], we did not include the biometrical relationship computed from catches during the reproductive period (i.e. June to August). For each specimen, we measured the Standard Length (SL, cm) with a *Vernier* calliper (to the nearest 0.005 cm) and the Total Weight (TW, g) with a Mattler Toledo balance (to the nearest 0.1 g) and used these parameters to calculate the Body Condition Index (BCI): that is the conditional state of a fish for a given length [68], according to the following equation [68], [69]:
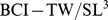



### Statistical Analysis

We tested differences in nautical traffic, measured as the number of moored boats, boat passages and their sum as total boat events, with an analysis of variance (ANOVA, [70]) treating area (2 levels: Area 1 and Area 2), period (2 levels: weekdays and holidays), and time of day (3 levels: morning, midday, and evening) as orthogonal and fixed factors. This analysis revealed three significantly different categories of traffic intensity (low, medium and high; see Results section) that were used in the following analyses.

We tested the effect of traffic intensity on the foraging rate of *C. chromis* with a Factorial ANOVA [71] by treating zone (2 levels: A-zone and B-zone) and traffic intensity (3 levels: low, medium and high) as fixed factors and the number of pecks min^−1^ as the dependent variable. We tested differences in the daily foraging patterns with a Factorial ANOVA [71] by treating zone (2 levels: A-zone and B-zone), period (2 levels: weekdays and holidays) and time of day (3 levels: morning, midday, and evening) as fixed factors and the number of pecks min^−1^ as the dependent variable.

We tested the effect of boats on school polarization times with a PERANOVA (i.e. a distance-based Permutational Analysis of Variance, [72]) by using type of boat presence (5 levels: no boat in A-zone, no boat in B-zone, moored boat, boat passage, and boat above) as independent factors. Conditions of no boats and boat passages above the school occurred while we recorded the school behaviour in both of our study areas, whereas moored boats and passages occurred only in one area. We then pooled polarization times from the two areas, having checked that they were not significantly different (ANOVA, Table S3).

We tested the effect of nautical traffic on school density with a PERANOVA (by using traffic intensity (4 levels: no traffic [absence of boat; i.e. A-zone], low [<40 boat h^−1^], medium [40< boat h^−1^<80] and high traffic [>80 boat h^−1^]) as the independent factor and school density - the number of individuals per video shot - as the dependent variable.

We tested differences in BCI between areas and zones with a Factorial ANOVA [71] with area (2 levels: Area 1 and Area 2) and zone (2 levels: A-zone and B-zone) as fixed factors.

Variables were squared or log-transformed when the assumption of homoscedasticity was violated (i.e. Cochran test p<0.05). If their distribution still presented heterogeneous variances after transformation, we lowered the significant value level from α = 0.05 to 0.01 [73].

## Supporting Information

Table S1 **Nautical traffic statistics.** Mean (±SE) number of boat passages, moorings and the resulting total number of boat events recorded in B-zones.
(DOC)

Table S2 **Pecking rate statistics.** Mean (±SE) pecking rate recorded within the A- and B-zones, during weekdays and holidays and in the three time slots.
(DOC)

Table S3 **Behavioural response to the same noise source in different areas.** Differences in the time of polarization events between the two study areas under the same type of boat presence. ANOVA allowed us to pool the data sets from the two areas.
(DOC)
