# Supplementary material for: Effects of Nautical Traffic and Noise on Foraging Patterns of Mediterranean Damselfish (Chromis chromis)
Source: PLoS One. 2012 Jul 11;7(7):e40582. doi: 10.1371/journal.pone.0040582 (PMC3394703; doi:10.1371/journal.pone.0040582)
Supplement: Table S1 — Nautical traffic statistics. Mean (±SE) number of boat passages, moorings and the resulting total number of boat events recorded in B-zones. (DOC) [file pone.0040582.s001.doc]

Table S1. **Nautical traffic statistics.**

|  | | | *Boat passages* | | | *Boat moorings* | | | *Total boat events* | | |
| --- | --- | --- | --- | --- | --- | --- | --- | --- | --- | --- | --- |
|  |  |  | Mean | ± | SE | Mean | ± | SE | Mean | ± | SE |
| B1 |  |  | 34.82 | ± | 3.14 | 19.73 | ± | 3.13 | 54.55 | ± | 5.47 |
| B2 |  |  | 25.57 | ± | 2.76 | 14.10 | ± | 2.57 | 39.67 | ± | 4.57 |
| weekdays |  |  | 17.35 | ± | 1.49 | 9.00 | ± | 1.36 | 26.35 | ± | 2.52 |
| holidays |  |  | 43.03 | ± | 3.23 | 24.83 | ± | 3.56 | 67.87 | ± | 5.63 |
| morning |  |  | 22.50 | ± | 2.45 | 9.33 | ± | 1.47 | 31.83 | ± | 3.51 |
| midday |  |  | 35.75 | ± | 4.15 | 28.28 | ± | 5.12 | 64.03 | ± | 8.04 |
| evening |  |  | 32.33 | ± | 3.96 | 13.15 | ± | 2.08 | 45.48 | ± | 5.40 |
| weekdays | morning |  | 14.55 | ± | 2.15 | 5.50 | ± | 1.71 | 20.05 | ± | 3.42 |
| weekdays | midday |  | 19.00 | ± | 2.63 | 14.15 | ± | 3.06 | 33.15 | ± | 5.07 |
| weekdays | evening |  | 18.50 | ± | 2.89 | 7.35 | ± | 1.70 | 25.85 | ± | 4.18 |
| holidays | morning |  | 30.45 | ± | 3.65 | 13.15 | ± | 2.10 | 43.60 | ± | 4.93 |
| holidays | midday |  | 52.50 | ± | 5.86 | 42.40 | ± | 8.78 | 94.90 | ± | 11.80 |
| holidays | evening |  | 46.15 | ± | 6.00 | 18.95 | ± | 3.36 | 65.10 | ± | 7.86 |
| B1 | weekdays | morning | 14.90 | ± | 2.88 | 7.70 | ± | 3.12 | 22.60 | ± | 5.66 |
| B1 | weekdays | midday | 20.90 | ± | 4.11 | 15.30 | ± | 5.90 | 36.20 | ± | 9.28 |
| B1 | weekdays | evening | 21.70 | ± | 4.26 | 9.60 | ± | 3.03 | 31.30 | ± | 6.77 |
| B1 | holidays | morning | 37.10 | ± | 5.12 | 17.10 | ± | 3.11 | 54.20 | ± | 6.33 |
| B1 | holidays | midday | 57.80 | ± | 8.49 | 45.50 | ± | 13.71 | 103.30 | ± | 18.06 |
| B1 | holidays | evening | 56.50 | ± | 7.12 | 23.20 | ± | 5.25 | 79.70 | ± | 10.07 |
| B2 | weekdays | morning | 14.20 | ± | 3.36 | 3.30 | ± | 1.21 | 17.50 | ± | 3.98 |
| B2 | weekdays | midday | 17.10 | ± | 3.39 | 13.00 | ± | 2.10 | 30.10 | ± | 4.53 |
| B2 | weekdays | evening | 15.30 | ± | 3.86 | 5.10 | ± | 1.39 | 20.40 | ± | 4.60 |
| B2 | holidays | morning | 23.80 | ± | 4.48 | 9.20 | ± | 2.34 | 33.00 | ± | 6.12 |
| B2 | holidays | midday | 47.20 | ± | 8.19 | 39.30 | ± | 11.63 | 86.50 | ± | 15.68 |
| B2 | holidays | evening | 35.80 | ± | 8.81 | 14.70 | ± | 3.99 | 50.50 | ± | 10.58 |

Mean (±SE) number of boat passages, moorings and the resulting total number of boat events recorded in B-zones.
