# Supplementary material for: Effects of Nautical Traffic and Noise on Foraging Patterns of Mediterranean Damselfish (Chromis chromis)
Source: PLoS One. 2012 Jul 11;7(7):e40582. doi: 10.1371/journal.pone.0040582 (PMC3394703; doi:10.1371/journal.pone.0040582)
Supplement: Table S2 — Pecking rate statistics. Mean (±SE) pecking rate recorded within the A- and B-zones, during weekdays and holidays and in the three time slots. (DOC) [file pone.0040582.s002.doc]

Table S2. **Pecking rate statistics.**

|  |  |  | Mean | ± | SE |
| --- | --- | --- | --- | --- | --- |
| A-zone |  |  | 11.64 | ± | 0.55 |
| B-zone |  |  | 18.32 | ± | 0.84 |
| weekdays |  |  | 17.09 | ± | 0.74 |
| holidays |  |  | 12.36 | ± | 0.66 |
| A-zone | weekdays |  | 11.40 | ± | 0.86 |
| A-zone | holidays |  | 11.84 | ± | 0.70 |
| B-zone | weekdays |  | 22.11 | ± | 1.07 |
| B-zone | holidays |  | 13.13 | ± | 1.25 |
| A-zone | weekdays | morning | 8.31 | ± | 0.98 |
| A-zone | weekdays | midday | 18.31 | ± | 1.47 |
| A-zone | weekdays | evening | 4.00 | ± | 1.06 |
| A-zone | holidays | morning | 10.07 | ± | 0.92 |
| A-zone | holidays | midday | 15.06 | ± | 1.39 |
| A-zone | holidays | evening | 10.71 | ± | 1.37 |
| B-zone | weekdays | morning | 26.09 | ± | 2.26 |
| B-zone | weekdays | midday | 22.29 | ± | 1.42 |
| B-zone | weekdays | evening | 19.41 | ± | 1.88 |
| B-zone | holidays | morning | 11.68 | ± | 1.47 |
| B-zone | holidays | midday | 6.69 | ± | 1.30 |
| B-zone | holidays | evening | 17.32 | ± | 2.47 |

Mean (±SE) pecking rate recorded within the A- and B-zones, during weekdays and holidays and in the three time slots.
