# Supplementary material for: Effects of Nautical Traffic and Noise on Foraging Patterns of Mediterranean Damselfish (Chromis chromis)
Source: PLoS One. 2012 Jul 11;7(7):e40582. doi: 10.1371/journal.pone.0040582 (PMC3394703; doi:10.1371/journal.pone.0040582)
Supplement: Table S3 — Behavioural response to the same noise source in different areas. Differences in the time of polarization events between the two study areas under the same type of boat presence. ANOVA allowed us to pool the data sets from the two areas. (DOC) [file pone.0040582.s003.doc]

Table S3. **Behavioural response to the same noise source in different areas.**

| Type of boat presence |  |  |  | ANOVA | | | |
| --- | --- | --- | --- | --- | --- | --- | --- |
|  | Mean | ± | SE | df | MS | *F* | p |
| no boat in A1 | 15.25 | ± | 2.74 | 1 | 438.79 | 3.9238 | 0.0588 |
| no boat in A2 | 23.45 | ± | 3.01 |  |  |  |  |
| Residuals |  |  |  | 25 | 111.83 |  |  |
| no boat in B1 | 41.62 | ± | 12.16 | 1 | 0.33 | 3.1191 | 0.0859 |
| no boat in B2 | 23.48 | ± | 3.56 |  |  |  |  |
| Residuals |  |  |  | 36 | 0.11 |  |  |
| boat passage in B1 | 63.33 | ± | 16.41 | 1 | 20.83 | 0.0110 | 0.8966 |
| boat passage in B2 | 67.5 | ± | 27.5 |  |  |  |  |
| Residuals |  |  |  | 3 | 1043.06 |  |  |

Differences in the time of polarization events between the two study areas under the same type of boat presence. ANOVA allowed us to pool the data sets from the two areas.
